# A Randomized Controlled Trial of Group Intervention Based on Social Cognitive Theory for Smoking Cessation in China

**DOI:** 10.2188/jea.17.147

**Published:** 2007-09-01

**Authors:** Pinpin Zheng, Fengxia Guo, Yue Chen, Yingying Fu, Tingting Ye, Hua Fu

**Affiliations:** 1 School of Public Health, Fudan University.; 2 Xuhui District Center for Disease Prevention and Control.; 3 Department of Epidemiology and Community Medicine, University of Ottawa.

**Keywords:** Smoking Cessation, Social Cognitive Theory, Randomized Controlled trial, Chine

## Abstract

**BACKGROUND:**

New training programs need to be developed to help Chinese smokers achieve quitting. The objective of this study was to assess the effectiveness of a group smoking cessation intervention based on social cognitive theory among Chinese smokers.

**METHOD:**

A total of 225 smokers were eligible for the study and were randomly assigned to an intervention group (n=118) and a control group (n=107). The intervention group received the course soon after a baseline survey, whereas the control group received routine training in the first 6 months, and then took the same course. Effectiveness was evaluated at 6-month and 1-year follow-up from baseline.

**RESULTS:**

After 6 months, 40.5% (47/116) in the intervention group and 5.0% (5/101) in the control group quit smoking (absolute risk reduction: 35.5% [95% confidence interval (CI): 24.2-46.8%]). The 6-month continuous abstinence rate was 28.4% (33/116) in the intervention group and 3.0% (3/101) in the control group (absolute risk reduction 25.4% [95% CI: 15.6-35.2%]). At 1-year follow-up, the proportion of quitting and the 6-month abstinence rate in the intervention group were 35.8% and 22.0%, respectively. The factors associated with smoking cessation during the 6 month period were intervention (adjusted odds ratio [OR]=6.42 [95% CI: 2.46-13.28]), as well as anticipation of quitting (adjusted OR=1.46 [95% CI: 1.12-1.91]) and skill self-efficacy score in the baseline (adjusted OR=1.04 [95% CI: 1.01-1.07]). The same intervention was conducted in the control group after the 6-month study, in which a similar intervention effect was observed.

**CONCLUSION:**

A smoking cessation intervention based on social cognitive theory among Chinese smokers is highly effective.

We know that tobacco dependence is a disease,^1^ and promotion of smoking cessation will reduce the burden of disease and improve population health.^2^ While efforts to promote smoking cessation need to be part of a much broader national tobacco control strategy that emphasizes prevention, it is clear that the greatest gains in reducing tobacco-caused morbidity and mortality in the next decades will come from helping addicted smokers to quit.^3^

Smoking cessation intervention in general includes pharmacological and behavioral intervention measures.^4^ Despite the effectiveness of the pharmacotherapies repeatedly confirmed by many studies,^5^ the high cost and unavailability in the clinical prescription become barriers of its application in China.

Behavioral interventions of smoking offer important alternatives to quitting on their own. Basically, they are the applications of different behavior theories. Motivational interviewing is a directive and client-centered therapeutic approach that intends to enhance patient's motivation to change through exploration and resolution of ambivalence.^6^ Even so, this advice appears to have its effect primarily by triggering a quit attempt rather than increasing the chances of success of quitting attempts.^7^ Another widely influential theoretical model that underlie smoking cessation is the transtheoretical model. However, forced smoking cessation such as hospitalization also makes identification of a stage of change confusing and results in high relapse.^8^ In recent years, social cognitive theory is well recognized as a useful framework for the design of smoking cessation intervention programs. The social cognitive theory is developed from the social learning theory, based on a dynamic and reciprocal model of interactions among behavior, personal factors, and environmental influences.^9^ According to the theory, behavior is a goal, and action outcome expectancies, self-efficacy and behavior capability as well as supporting environment are the core determinants for achieving the goal.^10^ The theory has been successfully applied in several clinical studies of smoking cessation intervention, especially for the patients of cancer or cardiac diseases.^11^^-^^14^

Till now, smoking cessation studies were mostly clinical based, with limited intervention studies focusing on community smokes.^15^^-^^17^ The interventions in community or workplace, with more people involved, would therefore have greater potential values to reduce rates of morbidity and mortality than that in clinical settings.^18^ There is a need to shift emphasis of smoking cessation intervention from narrow clinical approaches to more broadly approaches.

Quitting smoking is not common among Chinese smokers. It has been estimated that only less than 10% of them are presently trying to quit, and less than 4% of smokers are successful on smoking cessation for more than 2 years.^19^ In China, intervention measures mainly include propagandizing disease risks of tobacco use through media, creating smoking free settings in public places and providing advices for smoking patients in hospitals. Only a few published papers, however, have reported the effectiveness of smoking cessation intervention programs with ever fewer researches conducted in community.^20^^-^^22^

The present study was designed to assess the effectiveness and feasibility of a group smoking cessation intervention in community, through applying the social cognitive theory and comparing the proportion of smoking quitting between two groups with and without intervention.

## METHODS

### Study Setting and Design

The intervention study was carried out during the period from January 2004 through August 2005 in an urban community of Changqiao, Shanghai, with a population of around 103 thousand. It was a randomized, controlled trial with one intervention group and one control group.

### Participants

We worked with local organizations to recruit subjects through interpersonal communicating (persuading), advertising and broadcasting in the community. Eligible criteria for study participationwere: (1) being 18 years or older; (2) having smoked more than 100 cigarettes in life time and still smoking when they were recruited; and (3) willing to attend a five-session course and to be followed up for at least 6 months. Written informed consent was sought from all the participants. An approval for this study was obtained from the Ethics Committee of the School of Public Health, Fudan University.

The sample size was calculated for detecting the difference in rates of abstinence from smoking at 6-month follow-up between intervention group and control group.^23^ A previous study in China estimated a quitting rate of 9% in the control group and 30% in the intervention group.^19^ These estimates seemed realistic for the setting and public health perspectives. It was estimated that 72 subjects were needed for each group considering the alpha level of 5% and a statistical power of 90%. The final sample size was enlarged to 100 per group because some participants might withdraw from the study and covariates needed to be adjusted.

Once current smokers had consented to participate in the study, a baseline questionnaire investigation was conducted by a research assistant. Then, each individual was instructed to take one piece of folded paper out of a box with a mark of either "1" or "2" inside. Individuals who had a piece of folded paper marked "1", which they did not know before they took, were assigned to the intervention group and those had a piece of paper marked "2" were assigned into the control group.

**Figure 1.**
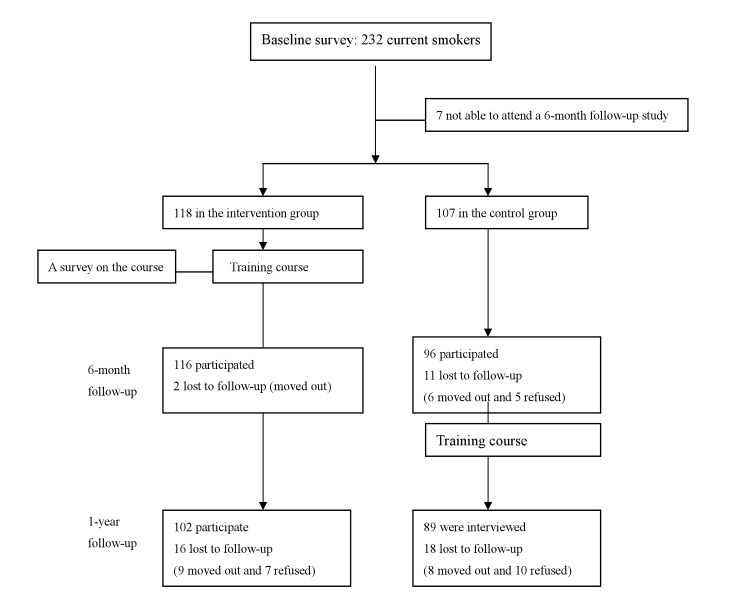
Flow-chart of the study design.

There were 118 smokers in the intervention group and 107 in the control group (Figure 1). Those in the intervention group received a 3-week training course of 5 sessions and provided their comments on the course afterwards. All participants were followed up, and information on their smoking habit, intention of quitting and self-efficacy in smoking cessation was again collected 6-month later. On the base of ethical consideration, the control group members were given brief advice to quit as soon as they finished baseline survey. They also received the same training course 6 months later. Both groups were followed up for 1 year after the intervention.

### Data Collection

The questionnaires covered smoking related information including questions on average daily cigarette consumption in the past week, age at smoking initiation, number of previous quit attempts, stage of change (intent to quit), self-efficacy in smoking cessation, perceived harm of smoking, extent of addiction, and self-belief in quitting ability.

Stage of change was assessed according to the trans-theoretical model of readiness for alteration^24^ and the readiness to change among smokers was divided into five stages including: (1) precontemplation (no intention to quit); (2) contemplation (cessation in the next 6 months considered); (3) preparation (intention of quitting in the next 30 days and steps taken to quit); (4) early action (cessation of smoking for less than 6 months); and (5) maintenance stage (cessation of smoking for 6 months or longer).

The extent of addiction was measured by six factors^25^ and the addiction score ranges from 0 to 10 by summing the six items, with a higher score indicating stronger dependence on tobacco. Smokers were considered to be highly addictive if they had a score of 6 or more. Moderate addiction and low addition were considered when smokers had a score of 4 or 5, and a score of 3 or less, respectively.

Self-efficacy (SE) in smoking cessation was assessed by SE scales.^26^ All SE items were measured on a 7-point scale and scored from "not at all sure I am able to" (−3) to "very sure I am able to" (+3). Emotional SE and social SE reflect the perceived SE with regard to engaging in new behavior in different situations; skill SE specifies the ability to use skills to cope with forces which contradict behavior change; relapse SE measures the extent of people's ability to maintain an attempt to change smoking behavior in spite of a relapse. Attempt self-efficacy is defined as perceived self-efficacy with regard to engaging partly or temporarily in new behavior.

Self-belief in quitting ability was measured as follows: "To what extent are you sure you can make a success in quitting?" A 7-point scale ranged from "not at all sure I am able to quit" (1) to "very sure I am able to quit" (7). General health status was measured by asking "In your opinion, how is your health? (a) excellent; (b) fair; or (c) poor".

The same questions on above information were used for the baseline survey and the 6-month and 1-year follow-up investigations. Demographic information was only collected at baseline, including age, gender, marital status, occupation, and educational level. The urine samples of participants were also collected from all participants after questionnaire in the 6-month and 1-year follow-up to confirm their smoking status by examining the cotinine levels. In addition, an anonymous self-administered questionnaire was completed by those in the intervention group after last session of the course, which provided comments and opinions on training method, curriculum content and time arrangement of the program.

A smoking quitter was defined by three parameters. First, the average number of cigarettes smoked during the past week was zero. Second, the subject was in early action or maintenance stage. The last, the cotinine level of urine displayed less than 25 ng/mL.^27^

A smoking quitter was further categorized as a continuous quitter for 6 months, when he/she showed in maintenance stage, which also matched the information from the telephone interview. Thus, the 6-month continuous abstinence rate was determined.

### Intervention Procedures

During the program training, 118 participants in the intervention group were divided into 8 subgroups according to their location in the community. Each subgroup was composed of 13 to 15 members. The intervention course comprised of five two-hour sessions, which were delivered by three health education professionals twice a week. In each session, there were 4 or 5 activities, using standardized teaching materials for both trainers and trainees. The first session discussed components of cigarettes and cigarette smoke, smoking associated with cancer and respiratory disease, how to prepare for quitting, and how to make a record of circumstances for each cigarette smoked. The second session covered information on tobacco associated with coronary heart diseases and stroke, benefits and difficulties of smoking quitting, some misunderstanding on smoking and quitting, the imagining relaxing method, how to make a plan of quitting smoking. In the third session, discussion focused on the detrimental effect of passive smoking, advantages versus disadvantages of smoking and quitting, gradual muscle relaxation, and some skills and tips in quitting. The fourth session consisted of calculating the expenditure on smoking, making a decision to be a quitter, and writing a farewell letter to cigarettes. In this session, we also invited some successful quitters to share their experience. The fifth session involved coping strategies in situation of having pressure to smoke, and how to prevent relapse. In addition, a small graduation ceremony was held in this session and each member who completed the curriculum was awarded a certificate and a glass marked with "I select no smoking". No adverse events were reported during intervention in both groups.

After the baseline survey, the smoking status of all subjects was followed up by another researcher through telephone during the following 12 months (4.2±2.5 times in the intervention group and 3.9±2.1times in the control group, p>0.05).

### Urine Collection and Analysis

The collection of urine samples took place after participants completed the questionnaire at each of the 6-month and 1-year follow-ups. Two urine specimens for each person were collected between2 and 3 o'clock in the afternoon, and were immediately frozen at −20°C before they were packed in dry ice and sent to the Department of Industrial Hygiene, School of Public Health, Fudan University for analysis. A high-performance liquid chromatography was used to measure the cotinine level, which has a detection limit of 0.32umol/L.^28^

### Statistical Analysis and Outcome Measure

The data were analyzed using the Statistical Package for the Social Science^®^, version 11.0. Fisher's exact test or chi square test was used to examine group differences for categorical variables, and Student t-test was used to examine differences between groups for continuous data. Logistic regression models were used to examine associations between various factors and smoking cessation at the 6-month follow-up after adjustment for age and stage of change at baseline. The intention-to-treat approach was used to calculate the proportion of quitting and the 6-month abstinence rate. Two analysis sets were made based on different assumptions.

Analysis Set 1: The subjects with missing data were classified into two categories: those who have moved to an untraceable address as well as subjects who decline to be involved in subsequent data. The subjects who moved out were excluded from the denominator for calculating while those who declined to be involved in follow-up survey were counted as smokers.^29^

Analysis Set 2: The analysis was made on the most conservative assumption in which all lost to follow-up subjects were regarded as smokers.

## RESULTS

### Baseline Characteristics of the Subjects

A total of 232 participants were enrolled in the studies from January through April 2004 and 225 members participated in the study. The average age of the intervention group was higher than that of the control group (56.4 vs. 53.2 years, p<0.05). Correspondingly, the average duration of smoking in the intervention group was significantly longer than that in the control group (31.6 vs. 28.3 years, p<0.05). The self-belief of quitting in the intervention group was higher than that of the control group (5.09±1.66 vs. 4.31±1.62). No significant differences were found in gender, education level, marital status, self-reported health, age of smoking initiation, daily cigarette consumption, extent of addiction, the number of previous quit attempt and perceived harm of smoking (Table 1).

**Table 1.** Baseline characteristics of the subjects.

Characteristic	Intervention	Control	p value
Mean age ± SD (year)	56.4±12.8	53.2±10.5	0.03
Male (%)	95.3	91.5	0.27
Higher education (%)*	7.0	9.3	0.47
Married status (%)	75.6	72.4	0.62
Self-reported health (%)			
Excellent	28.4	30.2	0.38
Fair	60.3	52.1	
Poor	11.3	16.7	
Three most frequently engaged occupations for the longest time			
Factory workers	35.1	31.4	0.42
Commercial and service personnel	15.0	12.9	
Officer	10.2	12.8	
Mean age at smoking initiation (year)	24.8±9.2	24.6±8.6	0.84
Mean duration of smoking ± SD (year)	31.6±14.1	28.3±10.3	0.04
Mean daily cigarette consumption ± SD (n)	15.4±11.4	14.9±7.5	0.71
The number of previous quit attempt	1.3±1.1	1.1±1.2	0.11
Extent of addiction (%)			
Low to middle	71.5	81.3	0.09
High	28.5	18.7	
Stage of change (%)			
Pre-contemplation	36.2	42.7	0.62
Contemplation	44.8	42.7	
Preparation	18.0	14.6	
Agree that smoking has done harm to health (%)	23.7	20.6	0.34
Self-belief in quitting ability	5.1±1.7	4.3±1.6	0.01

*: university degree or higher

SD: standard deviation

### Six-month Follow-up

In the 6-month follow-up study, 212 out of 225 smokers at baseline participated. A comparison of persons who did (212) and did not (13) complete the 6-month follow-up, there were no significant differences between two groups in average age, education, marital status and smoking status at baseline (p>0.05). The comparison of quitting rate between intervention and control group were made through intention-to-treat approach with different analysis sets. According to the analysis set 1, the proportion of smoking quitting was significantly higher in the intervention group (40.5%, 47/116) than in the control group (5.0%, 5/101) (absolute risk reduction: 35.5%, 95% confidence interval [CI]: 24.2-46.8%). Correspondingly, the 6-month continuous abstinence rate was 28.4% (33/116) in the intervention group and 3.0% (3/101) in the control group (absolute risk reduction: 25.4%, 95% CI: 15.6-35.2%). The result from the analysis set 2 showed that a quitting rate of 39.8% (47/118) in the intervention group, whereas that is 4.7% (5/107) in the control group (absolute risk reduction: 35.1%, 95% CI: 24.1-46.1%). And the 6-month continuous abstinence rate was 28.0% (33/118) and 2.8% (3/107) in the intervention and control group respectively (absolute risk reduction: 25.4%, 95% CI: 15.8-34.9%).

The average daily cigarette consumption was 3.9 (±5.7) in the intervention group, at 6 month and significantly lower compared with the control group, 13.1 (±6.8). According to the trans-theoretical model theory, it was estimated that in the intervention group 19.8% were in the pre-contemplation stage, 26.7% in the contemplation stage, 7.7% in the preparation stage, 12.1% in the action stage and 28.4% in the maintenance stage. In the control group, they were 49%, 35.4%, 8.3%, 2.2%, and 3.0%, respectively. Table 2 shows that the intervention group had a significantly greater decrease in daily consumption of cigarettes and greater increase in self-efficacy scores as compared with the control group.

**Table 2.** Changes in daily consumption of cigarettes and self-efficacy.

	Intervention	Control	p value
Average change of daily consumption ±SD	−11.51±9.84	−1.71±8.15	<0.01
Change of self-efficacy score* ±SD			
Emotional	5.75±11.40	−0.27±10.04	<0.01
Social	5.69±10.25	1.92±9.03	0.01
Skill	6.29±15.48	1.53±16.17	0.05
Relapse	3.52±11.46	3.20±11.30	0.76
Attempt	2.19±6.74	1.43±8.19	0.51

* : The change of a self-efficacy score was the difference between corresponding score at follow-up and the score at baseline.

Smoking quitting happened more frequently in the intervention group than in the control group (p<0.001). There were significant differences in anticipation of quitting and all SE scores at the baseline between the participants who quit at the 6-month follow-up survey and those who did not quit (p<0.05). Table 3 presents demographic and psychological factors associated with smoking quitting at 6-month follow-up in multiple logistic regression analysis. The results indicated that those in the intervention group were more likely to quit as compared with control subjects with an adjust odds ratio of 6.42. Anticipation of quitting and skill SE at the baseline were also significantly associated with quitting. Education, marital status, self-reported health and the extent of addiction were not significantly associated with smoking cessation.

**Table 3.** Factors associated with smoking quitting at 6-month follow-up.

Factors	AOR*	95% CI	p value
Intervention group vs. control group	6.42	2.46-13.28	0.001
Self-efficacy score at baseline**			
Emotional	1.01	0.96-1.04	0.91
Social	1.01	0.97-1.06	0.55
Skill	1.04	1.01-1.07	0.04
Relapse	1.03	0.99-1.07	0.2
Attempt	1.05	0.98-1.12	0.14
anticipation of quitting at baseline**	1.46	1.12-1.91	0.005
Extent of addiction: High vs. low or middle	1.15	0.48-2.76	0.76
Self reported health			0.91
Fair vs. excellent	0.9	0.40-2.02	0.8
Poor vs. excellent	0.77	0.23-2.60	0.37
Education			0.66
Senior high school vs. junior high school or lower	1.22	054-2.75	0.64
College or higher vs. junior high school or lower	0.57	0.12-2.69	0.48
Marital status			0.88
Widowed or divorced vs. married	1.01	0.33-3.01	0.99
Unmarried vs. married	0.75	0.29-2.10	0.61

* : adjusted for age and stage of change at baseline

** : treated as continuous variables

AOR: adjusted odds ratio

CI: confidence interval

### Course Evaluation in the Intervention Group

After the training course, individuals in the intervention group completed a self-administrated questionnaire anonymously. Of 118 subjects, 79.4% and 20.6% thought the training method "excellent" and "good"; 77.6% and 21.9% regarded the content as "very important" and "important"; 87.2% thought the time schedule was "appropriate"; and 74.6% expressed that they would mobilize other smokers to participate in the training course.

### Intervention in the Control Group

A total of 96 in the control group received the training course after the 6-month follow-up, and 89 were followed up for six months after they took the course. Thirty two (32/99, 32.3%) reported quitting and were verified by their cotinine levels. The 6-month continuous abstinence rate was 26.2% (26/99). Average tobacco consumption was reduced by 11.3 (±10.27) cigarettes/day.

### One-year Follow-up in the Intervention Group

A total of 102 in the intervention group participated in the 1-year follow-up study. Table 4 shows smoking status at baseline, 6-month follow-up and 1-year follow-up. One year after intervention, average cigarette consumption remained lower and several self-efficacy scores remained higher compared with baseline (p<0.05). The proportion of quitting and the 6-month continuous abstinence rate were 35.8% and 22.0% correspondingly, which showed no significant difference compared to those at 6-month follow-up. However, social-efficacy, skill-efficacy and relapse-efficacy scores decreased (p<0.05). (Table 4)

**Table 4.** Smoking status at baseline, 6-month follow-up and 1-year follow-up in the intervention group.

	Baseline	6-month follow-up	1-year follow-up
Mean daily cigarette consumption ± SD	15.39±11.35	3.92±5.65*	4.60±6.25*
Proportion of quitting (%)	0	40.5^‡,^* (39.8^§,^*)	35.8^‡,^* (33.0^§,^*)
Continuous abstinence rate of 6 month (%)	0	28.4^‡,^* (26.2^§,^*)	22.0^‡,^* (20.3^§,^*)
Stage of change (%)			
Pre-contemplation	36.2	19.8	28.4
contemplation	44.8	26.7	27.4
Preparation	18	7.7	6.8
Action		12.1	11.7
Maintenance		28.4	24.1
Mean self-efficacy score ± SD			
Emotional	−2.35±9.19	3.41±9.07*	2.21±9.79*
Social	−1.70±8.83	4.25±8.12*	2.51±9.23*^,†^
Skill	3.84±13.46	10.11±12.57*	6.30±15.32^†^
Relapse	3.06±9.77	6.58±8.99	4.49±11.35^†^
Attempt	2.36±5.46	4.55±5.21	3.25±1.47

* : Compared with baseline, p<0.05

† : Compared with 6-month follow-up survey, p<0.05

‡ : Analysis Set 1: The subjects with missing data were classified into two categories: those who have moved to an untraceable address as well as subjects who decline to be involved in subsequent data. The subjects who moved out were excluded from the denominator for calculating while those who declined to be involved in follow-up survey were counted as smokers.

§ : Analysis Set 2: The analysis was made on the most conservative assumption in which all lost to follow-up subjects were regarded as smokers.

## DISCUSSION

Our study showed that the group smoking cessation program based on the social cognitive theory increased the proportion of smoking quitting dramatically, with an adjust odds ratio of 6.42 in the intervention group compared with the control group at 6-month follow-up. Our results also showed a higher proportion of smokers' willingness to quit and lower daily cigarette consumption in the intervention group. The 6-month continuous abstinence rate at 1-year follow-up was still as high as 22.0% and the daily cigarette consumption remained low in the intervention group. In addition, the effectiveness of the training was also displayed in the control group and the 6-month continuous abstinence rate achieved 26.2%. It is worth mentioning that the statistical approach in this study was conservative; we used an intention-to-treat analysis which might underestimate the effectiveness. All these results indicate that the group cessation program based on the social cognitive theory is an effective and sustainable tool to help smokers abstinent from tobacco in Chinese urban residents.

In addition to the intervention, skill SE at baseline was also an important predictor of quitting. which was consistent with the finding from a previous study which showed that the better that smokers considered themselves to be able to execute the specified cessation skills (skill SE),the more often they made a quit attempt.^26^ Anticipation of quitting, a general judgement of a person's capability to achieve cessation, was also significantly associated with quitting. There was no significant association between the extent of addiction and behavior of quitting, which is in concordance with a previous report.^30^ Most importantly, smokers with a high addiction also benefited from the intervention with no pharmacotherapy, which would have an important public health implications in this county for the reason that nicotine replacement therapy and other pharmacologic aids are not popular because of high costs.

Self-efficacy is one of the core component of the social cognitive theory. It is increasingly recognized to be an important predictor of behavior change, including smoking status^8^^,^^30^ Our results showed that there were improvements in emotional and social self-efficacy scores after intervention. Several self-efficacy scores declined at 1-year follow-up, which poses a challenge for strengthening subsequent intervention training to maintain the abstinence of quitters.

In China, tobacco use has been strongly influenced by social convention and customs. The social conception that smoking is still seen as a normal and private behavior, demobilizes quitting attempts. In addition, lack of feasible supporting resources makes quitting more difficult for those who attempt to quit. As tobacco use is strongly influenced by social conventions, smoking cessation intervention should focus on changing norms of smoking. Our training course was designed to decrease social acceptability under Chinese culture, aiming to smokers with different education levels.

It is noticeable that result in our study is far more better as compared to that of a Meta-analysis of recent smoking cessation program in worksite (odds ratio=2.03).^31^ The quitting rate of this study is also higher than those of prior cessation programs based on social learning theory for patients in which the biochemical confirmed point quitting rate of 1-year follow-up varied from 15% to 32%.^11^^,^^12^^,^^14^ The probable explanation may be as following: Many research on smoking cessation intervention s were conducted in developed countries where the tobacco control policies have been well implemented and therapeutic approach is quit available. It is quite possible that early adopters of these treatment brought better result than later adopters, and that those smokers who were among the first try each of these treatments had higher self-efficacy on quitting. This is consistent with the fact that there was a significant decline in abstinence rates in recent years, about 10 percentage points, from over 40% to 30%.^32^ In countries where smoking cessation strategy has been engaged and many interventions have been conducted for a long time, people who still smoke are more likely the individuals whose smoking behavior cannot be easily changed through interventions. On the contrary, in China where smoking is popular and smoking cessation resource is quite lacking, a well designed, intensive behavior intervention may lead to better results among the smokers. The smoking cessation program may be adoptable in other countries where smoking rate is high and smoking cessation service is scarce. However, some modification is necessary to adjust the social and cultural background and make it applicable to local smokers.

The subjects of the study are those who were willing to attend a five-session course. Undoubtedly, five-session training is intensive and is not suitable for every person. Having no time to attend the full course is the most common reason for not participating in the study. Designing a more concise program may be a compromising strategy for the smokers with limited time. A worksite based smoking cessation program may also be considered.

The Reach, Effectiveness, Adoption, Implementation and Maintenance (RE-AIM) framework offers a comprehensive approach to considering five dimensions important for evaluating the potential impact of an intervention.^33^ When viewed from the RE-AIM perspective, the intervention may yield significant public health effects in China. The reach is very large because it was carried out in the communities and worksites where the smokers live or work. The high proportion of quitting in the 6-month and 1-year follow-up indicated the high efficacy in quitting. The adoptability is credible because the training is designed according to the Chinese culture and the participants with different education levels give high assessment to the course. Good implementation fidelity can be achieved because the training curriculum had been written in details, which ensure the adherence of the designed training. Lastly, the result of 1-year follow-up showed the maintenance of behaviour change. On the other hand, if the training curriculum can be engaged by community health practitioners, the program will be spread through institutionalization. Thus, the overall potential public health value (R×E×A×I×M) is expected to be large.

There are several potential limitations. First, in the designingprocess, to locate the subjects to the intervention and control groups, each participant was asked to take one out of two pieces of folded paper from a box, which marked "1" for the intervention group and "2" for the control group. Compared to using random number, this procedure allowed us to locate the participants in different time. Although we do not expect any selection bias related to this procedure, total randomness may be questioned. Second, the control subjects might be affected by the intervention to some extent. The subjects in both the intervention and control groups lived in the same community and some of them were neighbors and/or friends. This effect, however, should be in favor of quitting attempts in the control group and tended to result in an underestimation of the difference between the intervention and control groups. Third, since the intervention was also provided to the control group after 6 months, it was difficult to make a meaningful comparison between two groups at 1-year follow-up. Forth, most of the participants were in middle or old age in the community. We do not know if the training would work as well as among younger populations. Last but not least, although we designed this intervention based on the social cognitive theory and assessed its effectiveness, we did not measure all the changes in the theory construct. Further study should engage to explore possible mechanisms of such as intervention.

In conclusion, the smoking cessation intervention based on the social cognitive theory was an effective and feasible approach and could be applied to middle and old age smokers in urban China. Further studies should be needed to determine the effectiveness of the intervention in other populations.
